# Utilisation of High Molecular Weight and Ultra-High Molecular Weight Hyaluronan in Management of Glioblastoma

**DOI:** 10.3390/gels11010050

**Published:** 2025-01-08

**Authors:** Alex-Adrian Salagean, Cezara-Anca-Denisa Moldovan, Mark Slevin

**Affiliations:** 1 Department of Histology, George Emil Palade University of Medicine, Pharmacy, Science, and Technology of Târgu Mureș, 540142 Târgu Mureș, Romania; alex-adrian.salagean@umfst.ro; 2 School of Medicine, George Emil Palade University of Medicine, Pharmacy, Science, and Technology of Târgu Mureș, 540142 Târgu Mureș, Romania; moldovan.cezara@yahoo.com; 3 Center for Advanced Medical and Pharmaceutical Research, George Emil Palade University of Medicine, Pharmacy, Science, and Technology of Târgu Mureș, 540142 Târgu Mureș, Romania

**Keywords:** naked mole-rat, hyaluronan, glioblastoma, targeted drug delivery

## Abstract

HA (hyaluronan) has been considered in recent years as a naturally occurring modifiable gel-like scaffold that has the capability to absorb and release drugs over an extended period of time making it suitable as a potential chemotherapeutic delivery agent. Considering the limited treatment options available in the treatment of glioblastoma, in this review, we discuss the novel utilisation of ultra-high molecular weight HA—originally identified as a mechanism for maintaining longevity in the naked mole-rat—as both a protective and extracellular matrix-optimizing colloidal scaffold, and a means to deliver therapy in resected brain tumours. The unique properties of this unique form of HA cross-linked gel indicate potential future use in the prevention and treatment of both proliferative-based and inflammation-driven disease.

## 1. Introduction and Background

Glioblastomas (GBMs) are the most common malignant brain tumours, constituting 16% of all primary central nervous system (CNS) malignancies. They can emerge de novo or through the malignant transformation of more benign tumours, primarily affecting the brain, with 61% located in the frontal, temporal, parietal, and occipital lobes. Standard treatment involves surgical resection followed by radiotherapy and chemotherapy with temozolomide. The annual incidence is approximately 3.2 cases per 100,000 in the USA, according to the Central Brain Tumor Registry of the USA (CBTRUS 2016). Despite optimal surgical interventions and postoperative adjuvant therapies, GBMs remain almost invariably fatal due to their aggressive and invasive nature. The 5-year survival rate for GBM patients in the USA is about 5.5%, with a median overall survival (OS) of roughly one year. The median OS for patients undergoing gross total resection is reported to be 15.5 months, compared to 11.7 months for those with subtotal resection and 5.9 months for those without resection. Recent research indicates that GBMs may originate from various cell types with neural progenitor-like properties, ranging from neural stem cells to glial cells, each exhibiting different alterations in the signalling pathways. This finding challenges the previous belief that all GBMs arise exclusively from glial cells [1,2].

There remain significant challenges in GBM treatment including incomplete resection, significant genetic heterogeneity affecting response to treatment, the restrictive blood–brain barrier (BBB), and an immunosuppressive microenvironment. Glioblastoma is also known for its highly invasive and therapy-resistant nature, and although in the last decade, there has been a gradual increase in clinical trials testing new drugs, particularly those focused on immunotherapy and targeted therapies, there has been limited success to date [3,4].

Surgical resection and radiotherapy are standard procedures for glioblastoma treatment, but there are several options for chemotherapy. Temozolomide is considered the most effective, as it significantly improves overall survival (circa 3 months) when used in conjunction with radiotherapy. Other chemotherapeutic agents are available, although they may not provide the same level of efficacy as temozolomide. These alternatives are valuable, especially in regions where temozolomide is not available. Notable examples include Gliadel, which is a biodegradable implant that releases carmustine directly at the tumour site, and lomustine, commonly used in Europe as an alternative to bevacizumab, which is more frequently used in the US and Canada [5,6].

However, the primary challenge, regardless of the efficacy or mechanism of the selected chemotherapeutic agent, is its ability to reach the tumour site and then maintain an effective concentration. The BBB poses a significant obstacle. Various therapeutic strategies have been proposed to address this issue, including drug delivery using biological scaffolds (the subject of this review), and nanoparticle-based modulation, for example, using intravenously delivered ultrasound microbubbles, or optical enhancement to break down the BBB-associated tight junctions [7].

HA has gathered significant interest among researchers in recent years due to its unique hydrogel-like properties and capacity for drug absorption and release. Its prevalence in the extracellular matrix of the brain positions it as a highly attractive option for the creation of novel, modified scaffolds that could be used in drug delivery for enhanced targeting in brain cancer treatment.

## 2. Hyaluronan—More than a Naturally Occurring Scaffold?

HA is a polysaccharide prominently found in the ECM throughout the human body. Its molecular mass ranges between around 400 Da (one disaccharide), and 10 MDa, and its physiological properties are influenced by its polyelectrolyte and polymeric characteristics, as well as its viscous nature. Along with HA, the extra cellular matrix (ECM) also contains proteoglycans (versican, neurocan, aggrecan, etc.), link proteins and other glycosaminoglycans such as chondroitin sulphate, keratan sulphate and heparan sulphate. HA is composed of multiple units of D-glucuronic acid and N-acetyl-D-glucosamine to which different proteins and proteoglycans can attach, resulting in a three-dimensional (3D) network. HA is biocompatible and frequently interacts with various cell receptors, as a ligand for the CD44 glycoprotein and the receptor for HA-mediated cell motility RHAMM to facilitate cell communication and behaviour [8]. Moreover, HA can undergo a variety of relatively simple chemical modifications that enable cross-linking between polymer chains and the formation of highly tuneable scaffolds [9,10].

Proteoglycans, (of which HA is a major component), are the most prevalent biomolecules within the brain ECM, and their molecular weight dictates their behaviour and properties [11]. Due to this abundance, HA is critically involved in modulating and maintaining balance within numerous processes, including cell migration, proliferation, differentiation, maturation of neural stem cell progenitors (NSCP) during brain development and repair, and other cellular behaviours [10,12].

The potential of HA for enhancing drug delivery is well-established and has been extensively explored. HA is characterised as a matrix suitable for controlled drug release and has already been widely used in various biomedical applications, where chemical cross-linking or conjugation with a range of bio-macromolecules, allows it to encapsulate different drugs, even at the nanoscale. For a review, see [13]. This capability is exemplified by the findings in wound healing/tissue protection and repair as described in the examples below.

### 2.1. Tissue Repair and Regeneration

Xu et al. assessed the chondrogenic potential of hybrid HA-based hydrogel particles containing varying amounts of heparin (HP), [synthesised by inverse emulsion crosslinking] and loaded with bone morphogenetic protein-2 (BMP-2) [14]. They showed in vitro that by varying the concentration of heparin within the gel, the storage and release rate of BMP-2 could be fine-tuned to optimally upregulate the release of murine chondrocyte-C3H10T1 cell differentiation markers including aggrecan, Sox-9 and collagen type-II. This type of novel scaffold could be utilised in supporting cartilage regeneration and repair in meniscal tear injuries, preventing the development of osteoarthritis, as has been partially demonstrated within animal models [15]. Moreover, in respect of CNS tumours, supplying concerted targeted drug release and/or promoting the natural immune response as was observed in gelatin-HA hybrids loaded with tumour antigen MAGE-A5. This effectively killed melanoma B16-derived tumours in a rabbit model and the rationale could legitimately be applied to glioblastoma therapy [16].

The treatment of infected wounds presents significant challenges, primarily due to the inherently unfavourable microenvironment and the prevalence of drug-resistant bacteria. To model these conditions, Huang et al. induced infected wounds in rats and treated them with hydrogels composed of carboxymethyl chitosan (CC) and aldehyde HA (AHA) combined with vancomycin and polylactide-co-glycolide (PLGA) microspheres. These novel drug-loaded hydrogels enabled effective wound healing through substantial granulation tissue formation, reductions in inflammatory markers at the wound site (IL-12, IL-1α, IL-10, and TNF-α), enhanced angiogenesis, and significant reductions in wound size [17].

Similarly, Deng et al. developed a multifunctional hydrogel by encapsulating ultrasmall silver nanoclusters (AgNCs; antimicrobial) and deferoxamine (DFO; angiogenic) within polydopamine-coated hollow mesoporous manganese dioxide nanoparticles (PDA/H-mMnO_2_; for controlled release). Using a rat model of infected wounds, they showed that in the presence of this gel, ROS was effectively converted to oxygen concomitantly with increased vascularization enabling effective healing, whilst terminating the original infection [18].

These results provide supporting evidence that combinations of materials and active molecules can be effectively integrated with HA scaffolds to provide targeted and regulatory benefits, directly influencing tissue protection and recovery after CNS injury or for example tumour resection, where hypoxia-associated neurovascular decoupling via haemorrhage or other BBB disruption is the major source of cell death and morbidity [19].

### 2.2. Neurological Protection/Regeneration

Implementing protective strategies within the CNS is challenging in part due to its limited response to treatment, and, to date, no effective therapies have been identified that can successfully functionally repair CNS injuries which therefore ultimately leads to impairment of neurological functions in corresponding sites of injury (e.g., traumatic injury or tumour formation with mass effect) [20]. HA hydrogels, however, have demonstrated success in treating spinal cord injuries (SCI) and stroke in experimental animals.

Mothe et al. utilised a hydrogel blend of HA and methyl cellulose (HAMC) injected with adult brain-derived NSPCs in a subacute rat SCI model. The HAMC hydrogel was covalently modified with recombinant rat platelet-derived growth factor-A (rPDGF-A) to promote oligodendrocytic differentiation. They showed a reduced cavitation, improved graft survival, increased oligodendrocytic differentiation, and preservation of peri-lesional host oligodendrocytes, indicating that HAMC-rPDGF-A could be a promising vehicle for cell delivery in spinal cord injuries [21].

Besides oligodendrocytes, other glial cells such as astrocytes have an important role in the formation of debilitating scar tissue that is present at the level of SCI and within the brain after injury. For neurons to heal, they need to penetrate through the scar tissue that develops around the injured area. High molecular weight HA, the major constituent of the ECM of the CNS, may be protective in this regard.

Khaing ZZ et al. [22] showed that high molecular weight HA was able to lower the total number of glia/astrocytes, decrease the amount of chondroitin sulphate proteoglycan (CSPG), and ultimately protect the lesion against gliosis-mediated scar formation in a rat model of dorsal spinal hemi section, suggesting a direct protective capability and rationale for its application as a stabilizing matrix following brain or other CNS tissue disruption.

Moshayedi et al. used an HA hydrogel to deliver human neural progenitor cells (iPS-NPCs) transplanted into a mouse cortical model of photothrombotic stroke. The optimised HA hydrogel significantly extended the survival time of iPS-NPCs and influenced cell fate, by promoting glial, neuronal, and stem cell progenitor phenotypes. The fate of the stem cells within the hydrogel was tracked in vivo using MRI and confirmation was made that the system was effective in selectively controlling stem cell survival and differentiation, indicating it could be used as a therapeutic adjunct that could help to maintain tissue and cellular integrity within the brain after surgical intervention [23].

With this in mind, the potential capacity of HA matrices to support and protect the brain parenchyma needs to be considered in greater detail, including the characterisation of the associated signalling mechanisms, interactions with the major cell receptors, and particularly taking into account any potential adverse interactions that could actually promote tumour growth or stimulate neuroinflammation.

## 3. Hyaluronan in the Glioblastoma Microenvironment

The majority of interactions between GBM cells and HA within the brain ECM are mediated through interaction with its receptors which orchestrate the major vascular and tumour cell activity, therefore potentially representing a viable therapeutic target.

Whilst CD44 is the major cell surface receptor responsible for cell–cell interaction, cell adhesion and migration (and the main subject of this review), HA also binds to other relevant receptors such as the receptor for HA-mediated cell mobility, also known as RHAMM, located in the cell cytoplasm and exposed at the cell surface in response to injury or cytokines such as TGF-β [24]; and tumour necrosis factor (TNF)-stimulated gene-6 (TSG-6), a protein produced mainly by mesenchymal stem cells (MSC) [25], which is involved in both anti-inflammatory/tissue protective properties as a key molecule in disease pathology.

HA interactions with RHAMM in the CNS are primarily involved in supporting plasticity and remodelling after injury. For example, RHAMM-HA binding promotes astrocyte and microglia motility that is blocked by neutralizing anti-RHAMM antibodies and by peptides corresponding to the HA-binding domains of RHAMM [26,27]. In the injured brain there is an increase in low molecular weight HA production, e.g., after a stroke, and the cellular signalling pathways in neurons and microvessels address the remodelling process by stimulating angiogenesis and revascularisation, as well as the survival of susceptible neurons partly through interactions with RHAMM [28].

In tumourigenesis, the over-expression of RHAMM may be an indicator of adverse prognosis [29] and within the CNS, glioma stem cells (GSC), neural stem and progenitor cells (NSC/NPC) are amongst the most active in GBM, taking part in the development and differentiation of the tumour. RHAMM is highly expressed within NSC/NPC and has been shown to support the maintenance of GSC stemness, leading to a more aggressive tumour profile [30,31]. Maintaining the high molecular weight profile of native HA should therefore also protect against these phenomena but a detailed description of these mechanisms is beyond the scope of this review.

TSG-6, although constitutively expressed in some tissues, is typically upregulated in response to inflammation. TSG-6 then interacts with glycosaminoglycans (GAGs), including HA acting as an enzymatic catalyst, facilitating the covalent transfer of the heavy chain inter or pre-alpha inhibitor proteins from a chondroitin sulphate chain to its core structure; and where it may have a role in facilitating astrocyte-mediated glial scar formation within the spinal cord, and protecting against cognitive impairment following stroke or traumatic brain injury [32,33,34]. However, there is currently no evidence linking it to the pathological processes involved in GBM.

### 3.1. CD44 the Major Receptor for HA in Tumour Signalling

CD44, the major receptor for HA, is recognised as a marker of mesenchymal GSCs and the mesenchymal subtype of GBM, associated with poor prognosis and radiation resistance in human GBMs [35].

Xu et al. reported that CD44 knockdown via lentiviral shRNA reduced both tumour volume and proliferation rates in subcutaneous tumours of over-expressing U87MG/U251 glioma cell lines, while significantly inhibiting intracranial tumour growth and extending survival in these intracranial models [36]. In addition, when combined with standard GBM drugs (temozolomide and carmustine), CD44 depletion induced tumour cell apoptosis through the activation of caspase-3 and ROS-induced cytotoxic stress, culminating in a synergistic inhibition of intracranial tumour progression, further prolonging the median survival time of mice. This and other studies highlight the importance of understanding the CD44-HA interaction within GBM, since the application of HA scaffolds adjacent to these tumours could promote growth and invasive capacity.

Both HA concentration and its molecular weight are significant in this regard. For example, when patient-derived GBM cells were cultured in a 3D matrix with controlled HA levels, the. HA concentration was found to regulate cell invasion in a biphasic, patient-specific manner, probably at least partially dependent on the individual level of interaction with HA receptors on the recipient cells (possibly reflecting tumour sub-type and clinical outcome). This was further characterised by the identification of HA-phosphorylated ezrin linking HA-bound CD44 to the actin cytoskeleton, which when perturbed, reduced cell adhesion and blocked invasive capacity [37].

Hence, although targeting HA-CD44-ezrin complexes may block HA-mediated tumour cell invasion in the brain, we need to be sure that HA acting as a scaffold cannot contribute to this phenomenon. Modification (stabilisation and ultra-high molecular weight) of the HA 3D structure may provide the answer to this conundrum as we will see later in the review.

As a rule, high molecular weight HA is more stable, less active and tissue protective whilst low molecular weight or oligosaccharides of HA are highly angiogenic and pro-inflammatory. In this regard, Ooki T et al. demonstrated that, specifically, the high-molecular-weight HA plays a critical role in stimulating tumour-suppressive Hippo signalling pathways in breast epithelial cells. This tumour-suppressive effect was mediated through the clustering of the CD44 extracellular domain, a cell-surface glycoprotein known to interact with HA [38].

In this study (confirmed by others), the clustering of CD44 led to the recruitment of the polarity-regulating kinase PAR1b via the CD44 intracellular domain and subsequent disruption of the inhibitory interaction between PAR1b and the MST (mammalian Ste20-like kinase) complex, which is a core component of the Hippo pathway. By releasing MST from this inhibitory complex, high molecular weight HA promotes the activation of Hippo signalling, leading to the suppression of tumour growth and proliferation.

Conversely, low molecular weight HA exerts a pro-tumourigenic effect, competing with native-HA for binding to the CD44 receptor, thereby preventing the beneficial clustering of CD44 and enabling tumour progression by inhibiting Hippo signalling. This dual role of HA in tumourigenesis highlights the complex and context-dependent nature of its interactions with cellular signalling pathways in cancer development [36].

### 3.2. HA-Mediated Targeting for GBM

HA as a scaffold has great potential through chemical addition and modification to be utilised as a drug carrier and in the targeted sustained delivery of drugs and this could be of benefit in the treatment of GBM patients.

Lubanska et al. (2022), synthesised spherical diketopyrrolopyrrole-based conjugated polymer nanoparticles (CPNs) containing fluorescein-conjugated HA, (as a ligand for the CD44 receptor present on stem cell-like tumour initiating cells) in a patient-derived Zebrafish xenograft model of glioblastoma. They showed effective BBB permeability of this system and a concentration- and cell cycle phase-dependent selective uptake of HA-CPNs in CD44 positive GBM-patient derived cultures, with a concomitant decrease in cell stemness and capacity for invasion indicating potential use of this system as a therapeutic, but most importantly, providing a safe solution to the inclusion of HA without tumour-promoting effects [39].

Similarly, other studies have explored the therapeutic potential of HA-micelles (HA-M) as a drug delivery system, specifically encapsulating a 1:1 molar ratio of lauroyl-gemcitabine (Gem-C12) and honokiol (HNK). HA-M bound to the CD44 receptor, (its over-expression being strongly associated with poor prognosis in GBM), effectively facilitating receptor-mediated endocytosis delivering the chemotherapeutic drugs into the cells of U87 spheroids resulting in apoptosis. This targeted delivery enhanced the micelles’ penetration into dense tumour spheroids, also leading to improved survival rates in mice bearing orthotopic xenograft glioblastomas as a proof-of-concept model of GBM [40].

**Figure 1:**
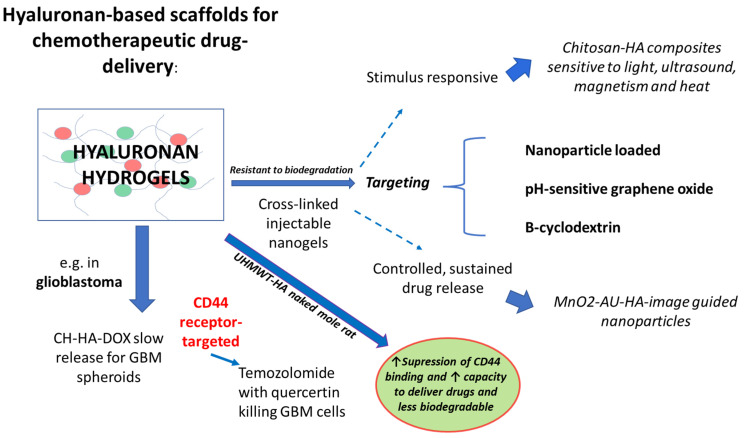
HA-based scaffolds for chemotherapeutic drug delivery: Shows a schematic of recent advances in the use of high molecular weight-HA for enhanced targeted drug delivery in cancer. HA hydrogels modified with chitosan (CH-HA) created a slow-release drug-hybrid system that secreted doxorubicin (DOX) and killed GBM cells [41]. Similarly, a combination of temozolomide with quercertin in a HA gel was effective in killing GBM cells in a CD44-dependent mechanism [42] More complex modifications have been tested primarily in vitro to date, that impart sensitivity to pH, magnetism, light and ultrasound, creating an active drug-delivery system via incorporation into the cancer cell membrane through specific receptors including CD44 (see ref [43] for a thorough review). All of these systems are targetable, with nanoparticle-based theranostic formulations being image guidable as demonstrated with magnesium dioxide (MnO2)-gold (AU)-HA [44] and other examples shown in [45,46,47]. The importance of HA-CD44 interaction should not be underestimated and suppression of CD44 binding remains as the cornerstone of tumour inhibition during utilization and optimization of these novel GBM and other tumour regulating composite gels.

These findings underscore the potential of HA scaffolds for enhancing drug delivery and efficacy in treating aggressive cancers like glioblastoma, where current treatment options are limited. The importance of stabilisation (e.g., through cross-linking) of such a 3D hydrogel is paramount to avoid hyaluronidase digestion to pro-tumourigenic oligosaccharides, whilst retaining a self-regulating drug-releasing capacity. In addition, blocking the interaction of HA with CD44 would be a further requirement of the therapeutic gel to avoid Hippo pathway signalling. The current state-of-the-art regarding HA hydrogel manipulation and use in targeted drug delivery is shown in Figure 1.

## 4. The Potential of Ultra-High Molecular Weight [Naked Mole-Rat] Hyaluronan in Targeted Cancer Therapy

Whilst the utilisation of high molecular weight HA (around 2 MDa) appears successful, a remarkable species of rat, the naked mole-rat (NMR) is uniquely able to synthesize what has become known as ultra-high molecular weight HA, and this may hold the key to providing an optimal treatment strategy in GBM, as described below.

### 4.1. HA, the NMR and Extraordinary Consequence

The NMR is a remarkable species, notable for its adaptation to low oxygen levels and exceptional cancer resistance. Its subterranean lifestyle has led to elevated levels of high molecular weight HA in its tissues. This adaptation confers numerous benefits, including preserved skin elasticity, youthful appearance, accelerated wound healing, protection against oxidative stress, and resistance to cancer and arthritis. It lives up to 40 years (10 times longer than other rodent species)! [48].

The high molecular weight HA present in the ECM of the NMR brain appears to form three-dimensional folded structures that resemble the macroscopic configuration of the gyri and sulci observed in the human brain [49]. The NMR fibroblasts secrete very high molecular weight HA (6–12 MDa), almost five times larger than mouse HA (0.5–3 MDa) [50] and human (0.5–2 MDa) [51]. The three main synthases involved in the production of HA are HAS1, HAS2 and HAS3, all having distinct functionality and regulatory mechanisms, but in the NMR, their expression is much higher (particularly HAS3), ensuring no loss of concentration over time [52].

This characteristic of the naked mole-rat brain’s HA appears at least in part to provide resistance by these mammals to the development of various forms of cancer, potentially including GBM and other CNS tumours. One of the processes involved in the resistance to cancer developed by the NMR is contact inhibition, a key anticancer mechanism that halts the cell cycle upon contact between cells. In humans, this process is mainly regulated by the p27 cyclin-dependent kinase inhibitor. NMR exhibit heightened sensitivity to this process, known as early contact inhibition (ECI), which prevents cell proliferation even with minimal contact. The ECI is thought to be modulated by HA, as it represents a critical component of the ECM [50]. In the NMR, the cyclin-dependent kinase inhibitor acts as a secondary defence, only engaging if ECI fails, offering extra protection against excessive cell division [53].

### 4.2. Studies Implicating NMR-HA in Cancer Protection

Tian X et al. [50] demonstrated that the naked mole-rat has extremely high molecular weight HA compared to mice, concomitant with increased binding to the CD44 receptor. In addition, the expression of hyaluronidase is also lower, and concomitantly, transmembrane protein-2 (TMEM2) a specific hyaluronidase that breaks down HA in other rodents, is modified in the NMR, with amino acid substitutions that nullify its normal catalytic activity, thereby protecting against HA breakdown [54]. All these characteristics play a role in mediating the cancer resistance of the NMR. When compared with other rodents (rat, mouse, guinea pig) examined by immunohistochemistry, the NMR showed the highest levels of HA, the strongest signals being in the epidermis, renal glomeruli and lymph nodes, and that high molecular weight HA was dominant compared to low molecular weight HA, potentially accounting for anti-aging and anti-cancer effects [55].

Zhao Y et al. utilised genetically modified breast cancer cell lines, 4T1/BT549-nmrHas2 to over express ultra-high molecular weight HA in vitro with a molecular weight of around 6Mda (similar to the NMR). The accumulation of HA induced enhanced apoptosis and inhibited cancer cell proliferation in 2D and 3D spheroid cultures and blocked tumour formation in nude mice [56]. The presence of ultra-high molecular weight HA was found to induce higher expression levels of the tumour suppressor protein p53, which in turn promoted the expression of pro-apoptotic proteins, p21 and Bax, thereby facilitating apoptosis.

Similarly, Zhang Z et al. demonstrated cancer resistance and extended lifespan conferred by high molecular weight HA using C57BL/6 transgenic mice overexpressing the naked mole-rat HA synthase 2 gene (nmrHas2). These genetically modified mice exhibited increased HA levels in various tissues, reduced incidence of spontaneous (lymphoma) and induced (skin papilloma’s) cancer, extended lifespan, and improved overall health. The most notable changes were attenuated systemic inflammation across multiple tissues, and transcriptomic/epigenetic alterations associated with maintenance of a lower biological age, suggesting that the longevity mechanisms evolved in the naked mole-rat can be transferred to other species [57]. It is worthy of note to mention that systemic lowering and avoidance of chronic inflammation may be one of the most critical factors protecting mammals from serious disease including cancer, cardiovascular disease and dementia [58].

The importance of the molecular weight of the HA to the overall physiological performance and health is in many ways astonishing, and this was reviewed eloquently by Michalczyk et al. (2023). Optimal modulation of the homeostatic balance of HA within an organism may provide a basis for ensuring stability within the ECM that enables normal cellular function with minimal stress [59]. Further studies are warranted to fully characterize this phenomenon and understand how to utilize NMR-HA in cancer and more specifically brain cancer and glioblastoma therapies.

Ultra-high molecular weight HA from the NMR has unique properties including a 3D conformational structure that mimics the gyri and sulci of the brain, imparting structural stability and superior retention capabilities acting as a cross-linked scaffold, in part mediated through the expression of TNFAIP6 which helps to prevent HA degradation [60]. The NMR HA appears to shield or block excessive CD44 binding and support normal cellular signalling through Src-MAPK, and unique Merlin/NF2-pALTINK4a/b novel signalling pathways suppressing tumour growth and invasion and instilling contact inhibition of cells [61]. Regarding repair and protection, the NMR-HA is strongly anti-inflammatory, inhibiting both IL1-β and TNF-α pathways and reducing potential scarring through the promotion of TGF-β signalling amongst others. Novel signalling through NMR interactions with HA are reviewed in detail by [49]. The unique characteristics of the HA from the NMR are highlighted in the green boxes. Abbreviations: TNFAIP6—tumour necrosis factor-inducible gene protein-6; pALTINK4a/b—pALT-inhibitors of cyclin-dependent kinase-4; HAS—hyaluronan synthase; FAK—focal adhesion kinase; HYAL—hyaluronidase; NF2—neurofibromatosis type 2; IL-1β—interleukin 1β; TNF-α—tumour necrosis factor-α.

## 5. Conclusions

**Figure 2:**
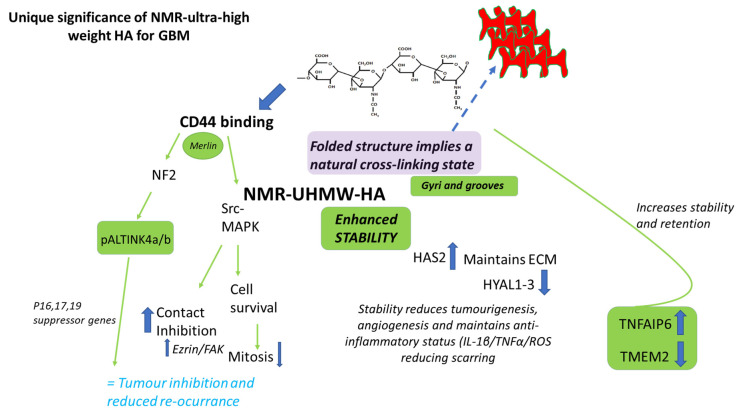
Unique significance of NMR-ultra-high weight HA for GBMs.

Ultra-high molecular weight HA may have enhanced stability and offer greater protection against chronic inflammatory conditions, providing a moderating scaffold that could also be suitable for extended drug delivery, for example, at sites of injury or for sustained chemotherapeutic treatment. The mechanisms by which it differs from regular native HA are primarily regulated by its interaction with the CD44 receptor, triggering distinct biological responses, which are also related to the ECI phenomenon observed in the NMR (see Figure 2 for details). Considering that in most mammalian species the average molecular weight of HA tends to decrease with age and yet the total amount remains consistent, a more detailed characterisation of the NMR- HA and its unique properties is required as it may lend itself to being the ideal scaffold for chemotherapeutic/combinational drug delivery in novel optimised or stratified GBM therapy.

## 6. Future Perspectives

Further studies are needed in order to understand, for example, how the NMR is able to synthesize and maintain such a high concentration throughout life, and what is required of the local micro-environment within the extracellular matrix in order to retain such an anti-ageing protective 3D milieu and how to optimize such a system for sustained, effective and targeted drug delivery in human cancer and other diseases.

## Data Availability

No new data were created or analyzed in this study.
